# Mechanistic Model of Alzheimer’s Disease Progression: Linking Amyloid and Tau Pathologies

**DOI:** 10.1002/alz.091295

**Published:** 2025-01-09

**Authors:** Stephen Duffull, Julie A Stone, Seth Robey

**Affiliations:** ^1^ Certara, Princeton, NJ USA; ^2^ Merck & Co., Inc., Rahway, NJ USA

## Abstract

**Background:**

Recent anti‐amyloid mAb trial results demonstrate slowing of Alzheimer’s disease progression, but to date do not fully halt or reverse this progression. Optimization of anti‐amyloid therapy (timing and duration of intervention, modality, combinations, biomarker guidance) is limited by incomplete understanding of the disease, such as relationship between amyloid and tau pathways. Mechanistic Alzheimer’s progression modeling investigated how amyloid and tau pathologies are connected in driving progression.

**Method:**

Calibration data (amyloid PET, CSF Ab42, CSF p‐tau, tau PET) were obtained from a wide‐range of publications including observational trials and interventional trials with BACE inhibitors, anti‐amyloid mAbs, and anti‐tau antisense oligonucleotides. First, mechanistic representations of the key features of amyloid (monomer, oligomer, plaque) and tau (tau, p‐tau, NFTs with spatial spread in brain) pathology were developed separately. Next a broad empiric exploration of a large range of amyloid‐tau links was undertaken with an objective to identify a limited number of models that can describe all the available clinical data.

**Result:**

Twenty eight variations on connecting models were considered; 12 were eliminated by review of their signature profiles, 3 due to redundancy of mechanisms, 6 due to implausible spatial mechanisms, 5 due to an inability to calibrate an amyloid component. The remaining 2 models (Amyloid plaque drives tau production or drives tau phosphorylation) could holistically describe all the available clinical data. Tau PET data from anti‐tau antisense trials were best described by a model with NFT loss (turnover 4‐times faster than amyloid plaque turnover). Further data is needed to fully characterize this process and related elements of the tau portion of the model. Simulations of early anti‐amyloid intervention (prior to emergence of tau pathology) suggested effects on progression would be enhanced relative to later intervention and emergence of tau pathology could be substantially delayed.

**Conclusion:**

Modeling which combines mechanistic representation of understood aspects of disease biology and empiric explorations of less well‐understood aspects and is calibrated to human trial data only can yield important insights. Such approaches have an important role to play in optimizing therapeutic strategies as the field moves into complex questions of combinations or sequencing of multiple therapeutic interventions.

